# Antibiotic prophylaxis for the prevention of infective endocarditis for dental procedures is not associated with fatal adverse drug reactions in France

**DOI:** 10.4317/medoral.22818

**Published:** 2019-02-24

**Authors:** Alexandra Cloitre, Xavier Duval, Sarah Tubiana, Pauline Giraud, Gwenaëlle Veyrac, Audrey Nosbaum, Aurore Gouraud, Julien Mahé, Philippe Lesclous

**Affiliations:** 1 DDS. Inserm, UMR 1229, RMeS, Regenerative Medicine and Skeleton, Université de Nantes, UFR Odontologie, CHU de Nantes, Service Odontologie Restauratrice et Chirurgicale, PHU4 OTONN, ONIRIS, Nantes, F-44042, France; 2 MD, PhD. Inserm, UMR 1137, IAME, Université Paris Diderot, Sorbonne Paris Cité, Inserm CIC 1425, Assistance Publique Hôpitaux de Paris, Hôpital Bichat Claude Bernard, Paris, France; 3 PharmD. Service de Pharmacologie Clinique, CHU de Nantes, F-44042, France; 4 MD, PhD. Inserm UMR 1111, CIRI, International Center for Infectiology Research, Ecole Normale Supérieure de Lyon, Université Claude Bernard Lyon 1, Allergy and Clinical Department, Université Lyon Sud, Hôpital Universitaire Pierre Bénite, Lyon, France; 5 PharmD. Regional Pharmacovigilance Center of Lyon, Pharmaco-Toxicology Department, Universitary Hospitals, Lyon, France; 6 DDS, PhD. Inserm, UMR 1229, RMeS, Regenerative Medicine and Skeleton, Université de Nantes, UFR Odontologie, CHU de Nantes, Service Odontologie Restauratrice et Chirurgicale, PHU4 OTONN, ONIRIS, Nantes, F-44042, France

## Abstract

**Background:**

One of the major reasons to stop antibiotic prophylaxis (AP) to prevent infective endocarditis (IE) in the United Kingdom but not in the rest of the world was that it would result in more deaths from fatal adverse drug reactions (ADRs) than the number of IE deaths. The main aim of this study was to quantify and describe the ADRs with amoxicillin or clindamycin for IE AP. The second aim was to infer a crude incidence of anaphylaxis associated with amoxicillin for IE AP.

**Material and Methods:**

The Medical Dictionary for Regulatory Activities (MedDRA) was used to group ADRs for IE AP using the broad Standardized MedDRA Queries “Anaphylactic reaction, Amoxicillin, Clindamycin, Clostridium Difficile infection” to the French Pharmacovigilance Database System. From this first-line collection, we selected all cases occurring for IE AP and ultimately, the cases for IE AP for a dental procedure. Then, each case was analyzed.

**Results:**

Of 11639 first-line recorded ADRs, 100 were for IE AP but no fatal anaphylaxis to amoxicillin or clindamycin and no *C. difficile* infection associated with clindamycin were identified. Only 17 cases of anaphylaxis to amoxicillin related to dental procedures were highlighted. The estimation of the crude incidence rate of anaphylaxis associated with amoxicillin for IE AP for invasive dental procedure was 1/57 000 (95% CI 0.2-0.6).

**Conclusions:**

Fatal or severe ADRs with amoxicillin or clindamycin is not a rational argument to stop IE AP before invasive dental procedures.

** Key words:**Infective endocarditis, antibiotic prophylaxis, anaphylaxis, adverse drug reaction, amoxicillin, clindamycin, pharmacovigilance.

## Introduction

Prevention of infective endocarditis (IE) is mainly based on antibiotic prophylaxis (AP) in patients with predisposing cardiac conditions undergoing invasive procedures responsible for bacteremia. This IE AP has been recommended since 1955 in the USA (1). Regularly updated over the past few decades, guidelines for IE AP are not concordant in every countries. In the United Kingdom (UK), the National Institute for health and Care Excellence (NICE) recommended complete cessation of any IE AP in any circumstances in 2008 until recently (2). In 2016, this institute amended its position, stipulating that in individual cases where the risk of IE posed to the patient is perceived as sufficiently high, or when patients themselves express a preference for it, AP may be appropriate (3). The rest of the world restricts systematic AP to patients with predisposing cardiac conditions at highest risk of IE undergoing the most invasive procedures (American Heart Association: AHA in 2007) (4); European Society of Cardiology: ESC in 2009, updated in 2015 (5); French National Agency for Drug Safety: ANSM 2011) (6). All agree on 3 highest-risk predisposing cardiac conditions (prosthetic valve, previous IE, cyanotic congenital heart disease) and AP regimen (a single 2-g amoxicillin oral dose 1 h preoperative, or a 600-mg oral dose of clindamycin in penicillin-allergic individuals, in adults). AHA also lists cardiac transplantation recipients who develop cardiac valvulopathy as high-risk of IE patients (4). The discrepancy between the British guidelines and the rest of the world is mainly based on the assessment of the benefit of IE AP.

The development of drug-resistant strains of oral bacteria, rare but potentially lethal drug reactions and more common adverse drug reactions (ADRs) such as gastrointestinal upset and the significant cost of antibiotics used for IE AP are claimed to outweigh the benefit of such AP (7). Even more, for the NICE, the risk that the number of deaths from anaphylaxis associated with IE AP, amoxicillin prophylaxis in particular, could exceed the number of deaths from IE that might be prevented by such IE AP (2). But data on fatal outcomes after IE AP seem to be very seldom.

In 2007, after oral intake for IE AP, the AHA was unaware of any cases of fatal anaphylaxis resulting from the administration of penicillin (including amoxicillin) recommended in the AHA guidelines for 50 years (4). Based on the Medicines and Healthcare products Regulatory Agency (MHRA) database, Lee and Shanson reported no fatal cases with the single 3 g oral dose of amoxicillin in the UK between February 1972 and May 2007 (8). However, other ADRs than fatal anaphylaxis are not documented in these papers. The NICE group, preparing clinical guidelines, reported that considering IE AP, no episode of anaphylaxis (whatever its degree of severity) to amoxicillin had been identified in the literature (2). Recently, also using the MHRA database between January 1980 and January 2014, no fatal reaction following a single 3-g oral dose used for IE AP was recorded (9). In addition, 67 non-fatal reactions, 16 of which were immune system disorders and 38 allergy-related skin disorders, were reported in the same period (9).

Data on severe or fatal outcomes after oral clindamycin intake for IE AP are also very seldom. One fatal case of documented *Clostridium difficile* colitis after a single dose of IE prophylactic clindamycin was published in 2001 (10). In the study of Thornhill *et al.*, 15 fatalities were recorded with a single 600-mg oral dose of clindamycin used for IE AP, mostly related to *C. difficile* infection (9). In addition, 178 non-fatal reactions, mostly gastrointestinal or allergy skin disorder reactions, were reported in the same period (9).

Except the case report of Bombassaro *et al.* (10), none of these papers focused on IE AP for invasive dental procedures as they also included IE AP for gastrointestinal, genitourinary or other interventional procedures. To date, no investigation of ADRs following oral administration of the two recommended antibiotics for IE AP, i.e., amoxicillin or clindamycin, specifically devoted to invasive dental procedures is available.

The first aim of this study was to quantify and describe anaphylactic reactions to amoxicillin or clindamycin and *C. difficile* infections related to clindamycin for IE AP for invasive dental procedures recorded in the French Pharmacovigilance Database System (FPDS) over a 31-year period. The second aim was to infer a crude incidence rate of severe or fatal ADRs associated with IE AP for invasive dental procedures correlating this number of cases to IE AP prescriptions in France over a similar period (11).

## Material and Methods

-Study design

The FPDS records all ADRs reported spontaneously by healthcare professionals collected by the 31 French regional pharmacovigilance centers since 1985 (12). This reporting is entirely voluntary. Every ADR report is analyzed by pharmacologists in the regional pharmacovigilance center. Causality is assessed for every suspected drug according to the French imputability method (13). ADRs are then registered in the FPDS and encoded according to the MedDRA classification.

-Data collection

First, we collected all anaphylactic reactions to both antibiotics, amoxicillin and clindamycin, recorded in the FPDS from September 1985 to July 2015 using the broad SMQ “Anaphylactic reaction”. SMQs are tools developed to facilitate retrieval of MedDRA-coded data as a first step in investigating drug safety issues in pharmacovigilance and clinical development. SMQs are validated, predetermined sets of MedDRA terms grouped together after extensive review, testing, analysis and discussion with experts (14). For this study, the diagnosis was established by applying criteria from the second symposium on the definition and management of anaphylaxis (15). According to this definition, anaphylaxis is a serious systemic reaction fulfilling at least one of the three following clinical criteria:

• Acute onset of an illness (within minutes to several hours) with involvement of the skin, mucosal tissue or both and respiratory compromise or reduced blood pressure or associated symptoms of end-organ dysfunction;

• Occurrence of two or more of the following signs, which occur rapidly, after exposure to a likely allergen for that patient (within minutes to several hours): involvement of the skin-mucosal tissues, respiratory compromise, reduced blood pressure or associated symptoms or persistent gastrointestinal symptoms.

• Reduced blood pressure after exposure to a known allergen for that patient (within minutes or several hours): infants and children: low systolic blood pressure (age-specific) or a > 30% drop in systolic blood pressure; adults: systolic blood pressure < 90 mmHg or > 30% decrease from that person’s baseline.

From this first-line collection, we selected all cases occurring for IE AP and ultimately, the cases for IE AP for a dental procedure. We classified all these reports according to the Ring and Messmer severity scale of anaphylactoid reaction ( Table 1) (16).

**Table 1:**
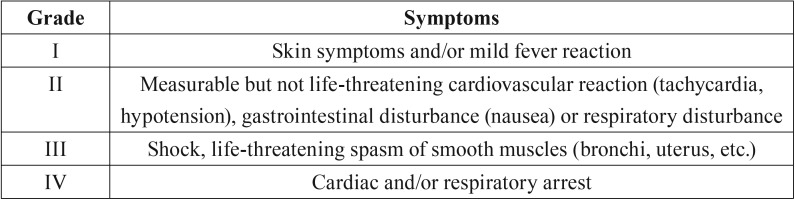
Severity scale of anaphylactoid reaction (according to Ring and Messmer) (16).

An additional request was made to extract cases of *C. difficile* infection related to clindamycin.

-Data analysis

For each case, the following data were collected: gender, age, clinical features, severity of symptoms, emergency treatment, possible risk factors of anaphylaxis, any associated medications and results of allergy testing (first-line tests: skin prick test (SPT) and intradermal test (IDT); second-line tests: specific IgE assay and basophil activation test (BAT) when available).

Descriptive statistics were performed using Microsoft Excel 2007 (Microsoft Corporation, France). We calculated numbers and percentages for each variable, mean ± standard deviation (SD). Statistical analysis was performed using the Student t-test. We considered *p*<0.05 to be statistically different.

## Results

-Case selection

**Figure 1:**
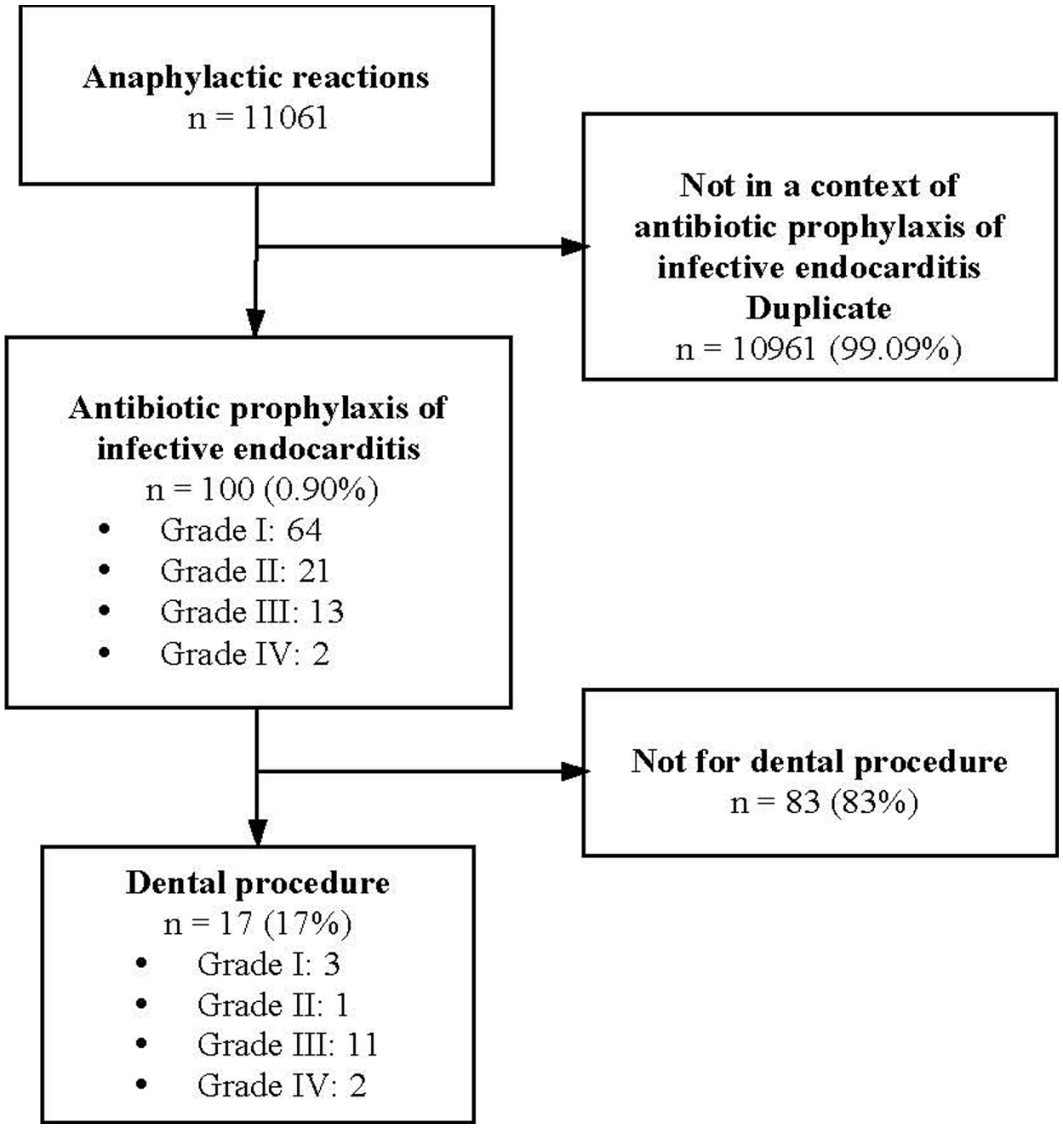
Flow chart of case selection for amoxicillin anaphylaxis. Grades of severity according to Ring and Messmer (16).

The first-line search identified a total of 11 061 cases of anaphylactic reactions to amoxicillin declared to the FPDS during this 31-year period by the broad SMQ “anaphylactic reaction”. This number decreased to 100 when targeting the selection on IE AP and after elimination of duplicates. Sixty four of them were classified as grade I, 21 as grade II, 13 as grade III and 2 as grade IV (Fig. 1). When restricting to dental procedures, this number fell to 17 cases (grade I: 3; grade II: 1; grade III: 11 and grade IV: 2) (Fig. 1). No case of fatal anaphylaxis with amoxicillin for IE AP was recorded for any type of procedure.

Using the same methodology, the first-line search identified a total of 536 cases of anaphylactic reactions to clindamycin. In addition, 42 cases of *C. difficile* infection were recorded when applying the supplementary request. However, when applying eligibility criteria – IE AP – no case of anaphylaxic reaction or *C. difficile* infection was selected.

-Demographics 

The age of the 17 selected patients varied from 25 to 85 years (49.43 ± 16.28); 10 were younger than 50 years old. No case was recorded in the young adult or pediatric populations. There was an overall female predominance (58.82%, *p*<0.01) ( Table 2). All the 17 cases selected were equally disseminated along the 31-year research period. Fifteen of them were patients with prosthetic heart valves and 2 patients had experienced previous IE.

**Table 2:**
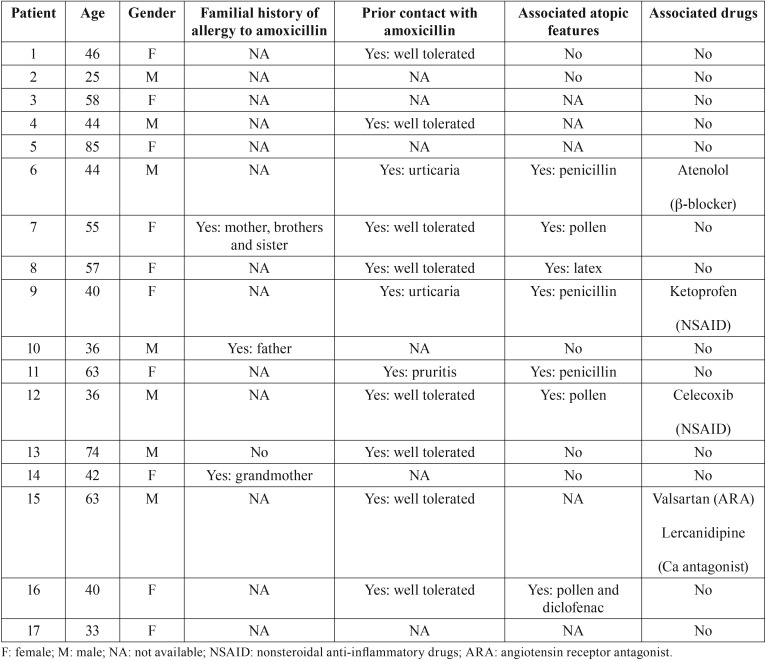
Demographic characteristics and allergy history.

-Clinical features

According to the Ring and Messmer classification, 3 of these 17 cases of amoxicillin anaphylaxis was classified as grade I because only affected by skin symptoms, 1 as grade II because affected by respiratory disturbance, 11 as grade III because having exhibited shock or life-threatening spasm of smooth muscles (mainly bronchi) and 2 as grade IV because cardiac arrests were reported. All the patients were hospitalized in an emergency department but only two of them were directly admitted in an intensive care unit. Corticoids, mostly intravenous prednisolone, associated or not with antihistaminics, were used in the majority of the cases and intramuscular epinephrine in the two most severe cases ( Table 3). The outcome of these 17 cases was complete recovery. Associated drugs that were potential risk factors enhancing the severity of anaphylaxis were recorded in four cases, NSAIDs (two cases), ß-blockers (one case) and an association of angiotensin II receptor antagonist with calcium antagonist (one case) ( Table 2).

**Table 3:**
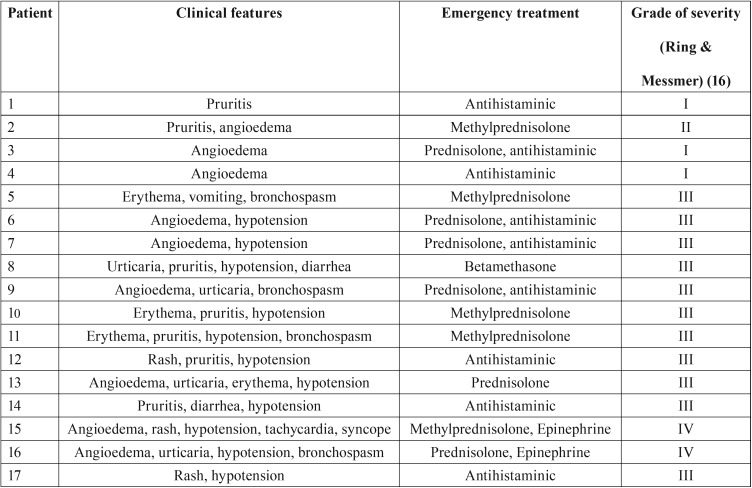
Amoxicillin anaphylaxis characteristics.

Various clinical features were observed ( Table 3). Cutaneous symptoms (urticaria, erythema, angioedema) were present in all but one case. Cardiovascular symptoms such as hypotension or collapse (but no cardiac arrest) were reported in 11 cases. Respiratory disturbances, mainly bronchospasms (but no respiratory arrest), were reported in 4 cases.

-Allergy history

Comorbidities such as atopic eczema/dermatitis, allergic seasonal rhinitis and asthma were reported in seven cases ( Table 2). No food intolerance was recorded. One case of cross-reactivity with diclofenac was recorded (case 16). Interestingly, 11 patients reported a history of previous medication with amoxicillin, seven of which had had good tolerance ( Table 2).

-Allergy tests

First-line skin tests, SPT or IDT, for amoxicillin were available for 14 of 17 patients ( Table 4). The SPT was positive in seven cases and IDT in four cases. A specific IgE assay was performed in eight patients and three were positive. In patients with negative specific IgE assays, the diagnosis of non-IgE-mediated hypersensitivity reaction was established. A BAT was available in four cases and positive in two of them.

**Table 4:**
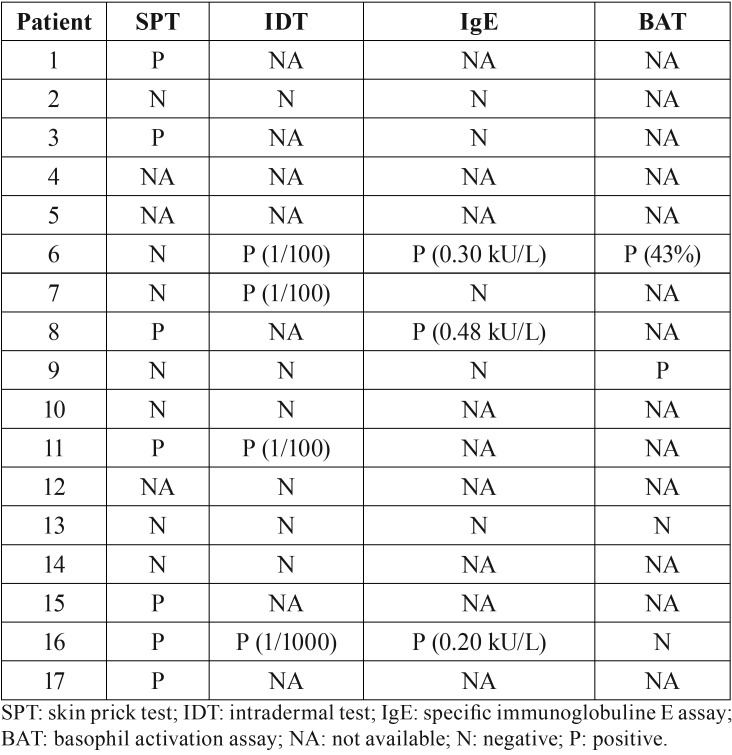
Allergy tests.

-Estimation of crude incidence rate of anaphylaxis associated with IE AP for invasive dental procedures

In a recent French crossover study, 138 876 patients with prosthetic heart valves (285 084 person-year) were included from January 2006 to December 2014 with a median follow-up of 1.7 year (11). During this 9-year period, 103 463 invasive dental procedures of which 52 280 (50.1%) were covered by IE AP which represent a total of 276 776 (1.7/9 x 52 280) invasive dental procedures if each of these patients would have a 9 year follow-up. Only 82.2% of these procedures were covered by amoxicillin IE AP which represent a total of 227 510 procedures. During the same period, 4 of the 17 selected cases in FDPS were recorded, all of them with amoxicillin in patients with prosthetic heart valves, so that the estimation of a crude incidence rate of amoxicillin anaphylaxis for IE AP for invasive dental procedures is 4//227 510 and so 1/57 000 (95% CI 0.2-0.6).

## Discussion

The main result of this study is the lack of fatal anaphylaxis with amoxicillin or clindamycin and *C. difficile* infections with clindamycin related to IE AP over a long 30-year period in France. Moreover, in patients with prosthetic heart valves undergoing dental procedures, the estimation of the risk of developing an anaphylaxic reaction with amoxicillin in this IE AP indication (1/57,000) appears lower than the risk of developing IE (1/46,000) (17).

In 1984, Bor and Himmelstein calculated a quantitative estimation of the risks and benefits of IE AP using penicillin for patients with mitral valve prolapse undergoing a dental procedure on the basis of published data, and the response to a questionnaire concluded that risks of IE AP outweighed its benefits (18). However, this conclusion cannot be worthy of consideration today since mitral valve prolapse is not a PCC at high risk of IE and penicillin G or V is no longer recommended for IE AP. Devereux *et al.* estimated that fatal allergic reactions to oral amoxicillin for IE AP in patients with mitral valve prolapse occurred with a frequency of 0.9 per million patients for IE AP (19). However, this estimation, derived from a study by Clemens and Ransohoff (20), was more related to penicillin than amoxicillin, considering dose, duration and route of administration. Once again, it was in patients for whom IE AP is no more required according to the ESC current guidelines.

Considering IE AP using only oral amoxicillin, fatal anaphylaxis has not yet been reported in the literature in either the USA (4), or the UK (8,9), or elsewhere in the world. Indeed, the AHA has failed to report fatal anaphylaxis associated with IE AP in the USA for the last 50 years where amoxicillin has been the mainstay of the recommended regimens (4), even if it was estimated a few decades ago that 1.36 people per million are likely to die annually from penicillin anaphylaxis to prevent IE, whereas not more than 0.26 annual deaths per million population are traceable to IE from dental procedures (21). This does not support the estimation of Seymour *et al.* that patients receiving penicillin (amoxicillin) prophylaxis to prevent IE are five times more likely to die from an anaphylactic reaction to the drug than to die from IE contraction (22). In a recent study also using the MHRA database, 73 fatal and 3072 nonfatal reports related to all doses, durations and routes of administration of amoxicillin as a single active constituent were reported from July 1963 to August 2014 (9). During the data-recording period from January 1980 to January 2014, this study did not reveal fatal reaction to a single 3-g oral dose of amoxicillin (dosage schedule and route of administration almost exclusively used for AP purposes) but revealed 67 nonfatal reaction reports (9). Consequently, the fatal and nonfatal ADR rates per million amoxicillin prescriptions were 0 and 22.6, respectively, which is very low, as also illustrated by the 1/57,000 estimation of the crude incidence rate of amoxicillin anaphylaxis in the present study for invasive dental procedures. This estimation should be compared to incidence rates for all-cause anaphylaxis ranging from 1.5 to 7.9 person-years in Europe (23), or 3.2 to 49.8 person-years worldwide (24). These incidences appear to be increasing in recent years and drug-induced anaphylaxis in particular. Ultimately, the AHA committee (and other agencies recommending IE AP for invasive dental procedures) believes that a single dose of amoxicillin is safe and is the preferred prophylactic agent for individuals who do not have a history of type I hypersensitivity reaction to a penicillin, such as anaphylaxis, urticaria or angioedema (4).

Regarding clindamycin, its association with *C. difficile* infection is well established even following a single dose administration (25). Only one case specifically related to the use of clindamycin for IE AP for a dental procedure has been reported in the literature (10). However, the occurrence of severe anaphylaxis to this molecule is thought to be rare (26). With a single 600-mg oral dose of clindamycin (dosage schedule and route of administration almost exclusively used for AP purposes), a UK survey based on the MHRA database from January 1969 to January 2014 revealed 15 fatal reactions (of which 12 were due to *C. difficile* infections) and 178 nonfatal reactions (one immune and 60 allergy-related skin disorder reactions) (9). In this study, the fatal and nonfatal ADR rates per million clindamycin prescriptions were 12.6 and 149.1, respectively, higher than those with amoxicillin (0 and 22.6, respectively) (9). In the present study, unlike Thornhil *et al.*, we recorded no fatalities associated with clindamycin for IE AP and many fewer nonfatal ADRs. However, it seems that the use of clindamycin for AP carries a significant risk of ADRs that need to be confirmed by additional studies. It must be remembered that clindamycin use for IE AP is only recommended as an alternative prescription in case of allergy to amoxicillin. Moreover, in France, clindamycin IE AP represents only 1% of the total IE AP, which probably minors the number of ADRs related to this molecule compared to what is observed in UK.

According to the current guidelines for management of anaphylaxis, epinephrine was used as emergency treatment in the most severe cases and a corticosteroid in the majority of the other cases (27). Cases with only cutaneous features were mainly managed with antihistaminic alone. Associated drugs, being potential risk factors enhancing the severity of anaphylaxis were recorded for four cases. Analysis of the reports highlighted comorbidity factors and particularly co-prescription of a NSAID, a well-documented family of drugs involved in anaphylactic reactions in such a way that the sole responsibility of amoxicillin in these reactions remains elusive.

Drug anaphylaxis is rarely documented by allergy tests, which may provide more information and thus prevent further reactions. Skin testing is the basic diagnostic tool but with several limits. Both SPTs and IDTs for amoxicillin were performed for the 15 patients of this study. Positive SPTs were associated with seven cases, whereas positive IDTs were recorded in four cases. Concordance between the two tests has only been observed in seven cases, two positive and five negative. This discrepancy is also recorded in a recent study investigating 333 cases of severe drug-induced anaphylaxis, of which 97 were related to amoxicillin. Only 50% of them showed a positive SPT, whereas 40.5% were associated with a negative SPT but a positive IDT (28). Moreover, skin test sensitivity for ß-lactams varied widely in different populations but also over the years. In a survey of 24 patients with a positive SPT to amoxicillin, only 50% of them were still positive after 1 year and none at 5 years, so that the efficiency of skin tests remains questionable for the diagnosis of anaphylaxis to amoxicillin (29). *In vitro* tests such as detection of specific IgE antibodies to ß-lactams in serum using immunoassays and detection on the basis of basophil activation are techniques considered to be complementary to *in vivo* tests, but in patients with severe anaphylactic reactions, the *in vitro* test should be the first choice in the diagnosis evaluation (29). This recommendation is probably too recent to be applied to all the patients of this study, but we noted that all the most recent patients except one were well documented. Hypersensitivity reactions to ß-lactams are classified as immediate when appearing within 1h of drug-intake and they are mediated by specific IgE-antibodies (30), and delated reactions are those occurring usually more than 1h after drug intake and are not IgE mediated (31). Moreover, both clinical patterns associating urticaria with angioedema (as recorded for patients 9, 13 and 16) are considered as more specific of an IgE-mediated reaction (31). However, the specific IgE assay was negative for patients 9 and 13, so that the sensitivity and/or the specificity of the tests calculated for these patients remain questionable. Taken together, these data highlight the need for future research to focus on optimizing diagnostic protocols and understanding anaphylaxis related to IE AP.

Our study has a number of unavoidable methodological drawbacks, as do most pharmacovigilance studies examining spontaneous notification. Reporting by health professionals is entirely voluntary and not all ADRs are reported, while those reported may omit relevant data (such as IE AP indication or route of administration), or may be cofounded by other factors. Many authors speculate that the prevalence of ADRs is underestimated from underreporting (32-34). In France, it has been estimated that less than 5% of all ADRs are spontaneously reported to the FPDS (32). But healthcare professionals are more likely to report a serious or fatal suspected ADR than a nonserious reaction. On another side, the reporting of ADRs for older drugs such amoxicillin is much lower than that for newer drugs (34). Consequently, based on available data, we can only speculate about the true incidence of antibiotic-induced ADRs, particularly anaphylaxis for IE AP.

## Conclusions

The NICE guidance and others suggest that the risk of fatal anaphylaxis from amoxicillin prophylaxis is so great that it would result in more deaths from anaphylaxis than the number of IE deaths prevented by giving AP. However, this fear is probably exaggerated, as there have been no reports of deaths from anaphylaxis following amoxicillin prophylaxis against IE, particularly related to invasive dental procedures, as shown by this study. IE AP with clindamycin could be a greater source of ADRs including fatalities. Unless convincing data are obtained from future research, consensus guidelines will continue to be based mainly on expert opinion rather than on hard evidence. This is not to say that fatal ADRs to amoxicillin and clindamycin does not exist, given the limitations of this study, but this argument cannot rationally be retained to stop IE AP. Ultimately, IE AP for invasive dental procedures appears to be safe and should be strengthened for predisposing cardiac conditions at high IE risk.
